# Comparison of a 10-Year Cumulative Age-Standardized Incidence Rate of Lung Cancer among Metropolitan Cities in Korea (During the 2000–2009 Period): Review of Occupational and Environmental Hazards Associated with Lung Cancer

**DOI:** 10.3390/ijerph15061259

**Published:** 2018-06-13

**Authors:** Joo Hyun Sung, Chang Sun Sim, Minsu Ock, Inbo Oh, Kyoung Sook Jeong, Cheolin Yoo

**Affiliations:** 1 Department of Occupational and Environmental Medicine, Institute of Health Sciences, Gyeongsang National University Changwon Hospital, Gyeongsang National University College of Medicine, 11, Samjeongja-ro, Seongsan-gu, Changwon-si 51472, Korea; yadaf@hanmail.net; 2 Department of Occupational and Environmental Medicine, Ulsan University Hospital, University of Ulsan College of Medicine, 877, Bangeojinsunhwando-ro, Dong-gu, Ulsan 44033, Korea; zzz0202@naver.com; 3 Department of Preventive Medicine, Ulsan University Hospital, University of Ulsan College of Medicine, 877, Bangeojinsunhwando-ro, Dong-gu, Ulsan 44033, Korea; ohohoms@naver.com; 4 Environmental Health Center, University of Ulsan College of Medicine, 877, Bangeojinsunhwando-ro, Dong-gu, Ulsan 44033, Korea; oinbo@naver.com; 5 Department of Occupational and Environmental Medicine, Hallym University Sacred Heart Hospital, 22, Gwanpyeong-ro 170 beon-gil, Dongan-gu, Anyang-si 14066, Korea; bandyoem@naver.com

**Keywords:** lung cancer, environmental hazard, occupational hazard

## Abstract

In Korea, lung cancer is a common cancer, and has the highest mortality rate in both males and females. Approximately 80% of lung cancer is due to smoking, and the remaining cases are known to be due to genetic factors, history of respiratory disease, infection, diet, and occupational and environmental factors. Since the occupational and environmental hazards may differ from region to region, the lung cancer risk may differ too. To identify this, we selected seven metropolitan cities, and compared occupational and environmental hazards. Furthermore, we calculated smoking rate adjusted standardized rate ratio (ratio of 10-year cumulative age-standardized incidence rate of lung cancer during the 2000–2009 period at target region versus reference region) to compare the regional lung cancer risk. The result showed that the emissions and concentrations of air pollutant were higher in high-risk regions, and the risk of lung cancer was significantly elevated in such area. In this study, we simultaneously consider the cumulative incidence, age-standardization and smoking rate adjustment. Therefore, we can conclude that the validity of the finding of this study is higher than that of past studies. In conclusion, the occupational and environmental hazards have an effect on lung cancer.

## 1. Introduction

In Korea, the Ministry of Health and Welfare started a cancer registry in 1980; they publish annual reports of cancer statistics, including the country’s cancer incidence data and the status of cancer occurrence throughout Korea. According to the annual report of 2015, lung cancer is the second most common malignant neoplasm in males, and the fifth most common in females [1]. It has the highest age-standardized mortality rate among malignant neoplasms in Korea [2]. The most common cause of lung cancer is smoking [3]. Studies based on international statistics suggest that approximately 80% of lung cancer is due to smoking [3,4], and in Korea alone, approximately 70% of lung cancers in adult males are due to smoking [5]. However, the remaining cases of lung cancer occur in non-smokers, and the likely causes are genetic factors, history of respiratory disease, infection, diet, obesity, occupational exposure to carcinogens, and air pollution [6]. Among these causes, occupational exposure to carcinogens and air pollution may differ by region, depending on the hazards of the region and occupational history. International studies have also reported differences in the incidence of lung cancer and mortality in different regions [7,8].

Occupational causes of lung cancer, as have been shown to be supported by sufficient evidence by the International Agency for Research on Cancer (IARC), include aluminum production, asbestos, non-ferrous industrial metals (arsenic, beryllium, cadmium, chromium (VI), and nickel), painting tasks, iron and steel founding, and radiation exposure [9]. Environmental causes include air pollution and atmospheric particulate matter (PM), which is associated with emissions from road traffic and industrial activities [9]. Therefore, workers who are exposed to these causes are at a higher risk of lung cancer. In addition, high levels of these emissions put those living near industrial complexes at higher risk for lung cancer compared to other areas [10,11]. Studies show that the concentrations of heavy metals in the blood of residents near industrial complexes are high [12,13], and therefore the concentration of other harmful substances is also likely to be high. Furthermore, additional unidentified causes of lung cancer may also affect nearby residents.

To confirm this, we first selected seven metropolitan cities in Korea in which a National Industrial Complex (NIC) is located, and examined the major industries in each region [14,15] (Table 1). The NICs refer to the industrial complexes designated by the government to promote key industries in Korea. In addition, data on the 5-year cumulative age-standardized incidence rate (ASR) of lung cancer (during the 1999–2003 period, 2004–2008 period, and 2009–2013 period) provided by the Korea National Statistical Office (KNSO) [2] was reviewed to compare the incidence rates of lung cancer among metropolitan cities (Table 2). During the period, the ASR of lung cancer in males was the highest in Ulsan. Onsan NICs (major petrochemical industries, non-ferrous metal industries, and iron and steel founding industries) and Ulsan/Mipo NICs (shipbuilding and automobile manufacturing) are all located in Ulsan.

As such, Ulsan has more industrial complexes than other cities, and these industrial complexes have a high proportion of painting, non-ferrous metal, iron and steel founding-related work. This type of work, according to the IARC, is associated with the development of lung cancer. Moreover, since petrochemical plants process and produce a variety of chemicals, air pollutant levels may be elevated; therefore, workers are exposed to these substances, putting them at risk for lung cancer. Therefore, it is possible that the higher incidence of lung cancer in Ulsan is associated with occupational and environmental causes.

However, the data provided by the KNSO does not consider smoking rates. Also, by only using the NIC data, we cannot confirm the association between lung cancer and occupational/ environmental causes. In this study, we will compare the incidence of lung cancer in seven metropolitan cities of Korea based on data of the cancer registry of 2000–2009, taking into consideration smoking rate and the air pollution index in the target area.

## 2. Materials and Methods

### 2.1. Study Population

The Korea Ministry of Health and Welfare started a nationwide cancer registry, the Korea Central Cancer Registry (KCCR), in 1980. Currently more than 190 hospitals participate in the KCCR, and the database covers more than 90% of the new cancer cases in Korea. The KCCR has constructed the Korea National Cancer Incidence Database (KNCIDB), which is well organized; it is easy to access patient information using the resident registration number (RRN) [16]. In Korea, the RRN consists of a 13-digit number given at birth and includes data on birth date (the first six digits), sex (the seventh digit), and birthplace, and it varies from person to person. The RRN can be used to identify a person throughout their life in many fields such as financial transactions and employment in Korea.

In order to examine the association between occupational/environmental hazards and lung cancer, we selected subjects from the KNCIDB who were classified as C33 (those with a malignant neoplasm of the trachea) and C34 (those with a malignant neoplasm of the bronchus and lung) according to the criteria of the International Classification of Disease-10 (ICD-10), from the country’s entire population and seven metropolitan cities from 1 January 2000 to 31 December 2009.

Finally, 165,894 subjects (120,265 males and 45,629 females) were selected from the entire country. By region, there were 27,442 subjects (19,291 males and 8151 females) in Seoul, 11,703 (8425 males and 3278 females) in Busan, 8213 (5858 males and 2355 females) in Daegu, 7167 (5154 males and 2013 females) in Incheon, 3807 (2667 males and 1140 females) in Gwangju, 3834 (2770 males and 1064 females) in Daejeon, and 2746 (1982 males and 764 females) in Ulsan.

### 2.2. Air Pollution Exposure Assessment

Since 2001, companies in Korea have been obliged to report pollutant release and transfer register (PRTR) [17] information to the National Institute of Chemical Safety (NICS). In addition, Korea has installed a total of 504 air monitoring networks and atmospheric heavy metal monitoring networks in 93 cities, and air pollution levels are measured. These measurements are then reported to the National Institute of Environmental Research (NIER) [18].

The PRTR data includes information on the total amount of carcinogen emissions (human carcinogens, IARC group 1; probable human carcinogens, IARC group 2A; possible human carcinogens, IARC group 2B) associated with lung cancer. Although the PRTR data do not indicate the concentration of substances, they can be used as a reference for estimating air pollution indirectly because they provide information on the total annual carcinogen emissions by region. The PRTR data have been analyzed based on the data reported during the 2001–2009 period. We used this data to examine the total amount of carcinogen emissions and IARC group 1 substances (asbestos, arsenic, chromium (VI), and nickel), known as causative agents of lung cancer, in target regions.

The annual report of ambient air quality in Korea published by the NIER shows concentration trends of substances for specific periods of time, since NIER has continuously measured the concentration of air pollutants (sulfur dioxide, SO_2_; nitrogen dioxide, NO_2_; ozone, O_3_; carbon monoxide, CO; particulate matter 10, PM10; lead, Pb; cadmium, Cd; chromium, Cr; copper, Cu; manganese, Mn; iron, Fe; nickel, Ni) [18]. We used these data to compare the concentrations of air pollutants from 1998 to 2009 in seven metropolitan cities. Six substances (SO_2_, NO_2_, O_3_, CO, PM10, and Pb) were used to represent the average concentration of air pollutants in Korea, as only these substances could be collected in the affected period.

We used the average emission amounts and ambient concentrations of air pollutants obtained from the PRTR and NIER data, respectively, as surrogates for population exposure to urban air pollution. Considering that most of the study population in a specific city would be normally exposed to average pollutant concentration in that city, air pollutant emission amounts and average concentrations can reflect the overall population exposure to ambient air pollution.

### 2.3. Smoking Rate Assessment

In this study, we used the data of community health surveys (CHS) [19] to estimate the smoking rates of adult males and females in target regions, because the KNSO did not provide the smoking rate of females in target regions. Because CHS began in 2008, this was the earliest year from which we were able to obtain smoking rates. Thus, we used the last year of the study period (2009) to represent the smoking rate. We requested raw data from the Korean Centers for Disease Control and Prevention (KCDC) and calculated the smoking rates of males and females in seven metropolitan cities, and from the entire Korean population using these data. Subjects who smoked cigarettes during the investigation periods were classified as current smokers and others were classified as non-smokers.

### 2.4. Crude Rate, Age-Standardized Incidence Rate, Standardized Rate Ratio, and Adjusted Standardized Rate Ratio

Since KCCR does not provide raw data publicly, we requested from the KCCR information on cases, crude rate (CR), age-standardized incidence rate (ASR) of lung cancer, and the standard error of ASR, of seven metropolitan cities and the entire Korean population, pertaining to 1 January 2000 to 31 December 2009.

CR is defined as the number of new cancer cases in a particular population during the observation period. Generally, the number of cancer patients per 100,000 people is calculated as indicated in equation 1. Since the study period was from 2000 to 2009, the person-years of observation were calculated using the resident registration population (RRP) of 1 January 2000 to 31 December 2009, according to the KNSO, using Equation (2):

(1) $$\mathrm{Crude}\;\mathrm{rate}\;\left(\mathrm{per}\;100,000\;\mathrm{person}-\mathrm{years}\right)=\frac{\mathrm{Number}\;\mathrm{of}\;\mathrm{new}\;\mathrm{cancer}}{\mathrm{person}-\mathrm{years}\;\mathrm{of}\;\mathrm{observation}}\times 100,000$$

(2) $$\mathrm{Person}-\mathrm{years}\;\mathrm{during}\;\mathrm{the}\;2000–2009\;\mathrm{period}=\frac{\left(\mathrm{RRP}\;\mathrm{of}\;1\;\mathrm{Jan}\;2000\times \mathrm{RRP}\;\mathrm{of}\;31\;\mathrm{Dec}\;2009\right)}{2}$$

RRP: resident registration population

ASR is a weighted average of CR and calculated as indicated in Equation (3). ASR is useful in comparing cancer risk of certain regions when there is a difference in age structures. The weights used in the calculation of ASR were the proportions of people in the corresponding age groups of the standard population. In this study, we used the Korean standard population (year 2000) [1] which is suggested by KCCR as the standard population (Table 3).

(3) $$\mathrm{ASR}=\frac{\sum (\mathrm{crude}\;\mathrm{rate}\;\mathrm{for}\;\mathrm{age}\;\mathrm{group}\times \mathrm{standard}\;\mathrm{population}\;\mathrm{for}\;\mathrm{age}\;\mathrm{group})}{\mathrm{Standard}\;\mathrm{population}}$$

ASR: age-standardized incidence rate

Standardized rate ratio (SRR) is the ratio of standardized rates and is useful in comparing the risk of cancer between regions. In this study, we calculated SRR using the ASR value and the standard error of ASR of target regions provided by KCCR. We adjusted for the smoking rate to compare the risk of lung cancer among target regions more precisely.

### 2.5. Analytic Methods

To compare the concentration of SO_2_, NO_2_, O_3_, CO, PM10 and Pb according to the target region, we conducted analysis of variance (ANOVA) using IBM SPSS Statistics for Windows, version 24.0 (IBM, SPSS Inc., Chicago, IL, USA). 

We used Excel 2010 (Microsoft, Redmond, WA, USA) to calculate the smoking rate, SRR, and the smoking rate adjusted SRR.

#### 2.5.1. Smoking Rate

As in the case of ASR, the smoking rate used in this study was the weighted average smoking rate, calculated by weighting the ratio of the standard population of each age group. We calculated the smoking rate using Equation (4):

(4) $$\mathrm{Smoking}\;\mathrm{rate}=\frac{\sum (\mathrm{smoking}\;\mathrm{rate}\;\mathrm{for}\;\mathrm{age}\;\mathrm{group}\times \mathrm{standard}\;\mathrm{population}\;\mathrm{for}\;\mathrm{age}\;\mathrm{group})}{\mathrm{Standard}\;\mathrm{population}}$$

#### 2.5.2. Standardized Rate Ratio

In order to compare the risk of lung cancer among target regions, we calculated SRR which is the ratio of the ASR in this study. The value of ASR and the standard error of ASR of the target regions, which are provided by KCCR, were used in the calculation. We used the nationwide ASR as the reference, and the significance was tested using Smith’s method [20,21]. Using this method, we also calculated the 95% confidence interval (CI). If the intervals did not contain a value of 1.0, it meant that the SRR was significantly different. These values were calculated using Equations (5)–(8):

(5) $$\mathrm{SRR}=\frac{{\mathrm{ASR}}_{1}}{{\mathrm{ASR}}_{2}}$$

(6) $$\mathrm{Lower}\;\mathrm{limit}\;\mathrm{of}\;95\%\;\mathrm{Confidence}\;\mathrm{Interval}={\frac{{\mathrm{ASR}}_{1}}{{\mathrm{ASR}}_{2}}}^{1-(1.96/X)}$$

(7) $$\mathrm{Upper}\;\mathrm{limit}\;\mathrm{of}\;95\%\;\mathrm{Confidence}\;\mathrm{Interval}={\frac{{\mathrm{ASR}}_{1}}{{\mathrm{ASR}}_{2}}}^{1+(1.96/X)}$$

(8) $$X=\frac{{\mathrm{ASR}}_{1}{-\mathrm{ASR}}_{2}}{\sqrt{{{(\mathrm{Standard}\;\mathrm{error}\;(\mathrm{ASR}}_{1}))}^{2}-(\mathrm{Standard}\;\mathrm{error}\;\left({\mathrm{ASR}}_{2}\right){)}^{2}}}$$

SRR: relative risk of standardized rate for ASR_1_ (target area) versus ASR_2_ (Korea)
ASR_1_: age-standardized incidence rate of target area
ASR_2_: age-standardized incidence rate of reference area (Korea)

#### 2.5.3. Adjusted Standardized Rate Ratio

We used Axelson’s indirect method [22] to adjust for the smoking rate. This method was first introduced in 1988 and has been used in other studies to indirectly correct for confounding variables [23,24,25,26]. We used the value of 2.58, which is the relative risk of lung cancer for current smokers versus non-smokers in Korea, as reported by the National Cancer Center in 2014 [5] as the standard relative risk for current smokers in Korea. These values were calculated using Equations (9) and (10):

(9) $$\mathrm{aSRR}=\frac{\mathrm{SRR}}{{\mathrm{RR}}^{\prime }}$$

aSRR: smoking rate adjusted SRR
SRR: relative risk of standardized rate for ASR_1_ (target area) versus ASR_2_ (Korea)

(10) $${\mathrm{RR}}^{\prime }=\frac{{\mathrm{RR}\times \mathrm{CSR}}_{\mathrm{target}}{+(1-\mathrm{CSR}}_{\mathrm{target}})}{{\mathrm{RR}\times \mathrm{CSR}}_{\mathrm{Korea}}{+(1-\mathrm{CSR}}_{\mathrm{Korea}})}$$

RR’: relative risk of lung cancer for target area versus Korea
RR: relative risk of lung cancer for current smoker versus non-smoker in Korea
CSR_target_: Current smoking rate for target area in 2009
CSR_Korea_: Current smoking rate for Korea in 2009

## 3. Results

### 3.1. Air Pollution Exposure

From 2001 to 2009, the total amount of all carcinogenic substances emitted during the period was highest in the Ulsan area (Table 4). Asbestos was identified only in Daegu from 2001 to 2006, and arsenic was only identified in Ulsan from 2005 to 2009. Chromium air emissions were identified in all regions except Seoul. In Busan, air emissions of chromium have been reported from 2001 to 2009, and the emitted amount of chromium was the highest with an average of 751 kg/year during the entire period. Chromium emissions were the second highest in Incheon, but there were no chromium emissions prior to 2003 in Daegu, and Ulsan also had no air emissions before 2003. There were no chromium emissions in Daejeon before 2004 and in Gwangju before 2005. Nickel air emissions were identified in all areas except Daejeon. In Incheon, there have been air emissions of nickel since 2002, and the emitted amount of nickel was the highest of all regions with an average of 1865 kg/year during the entire period (Table 5).

**Figure 1:**
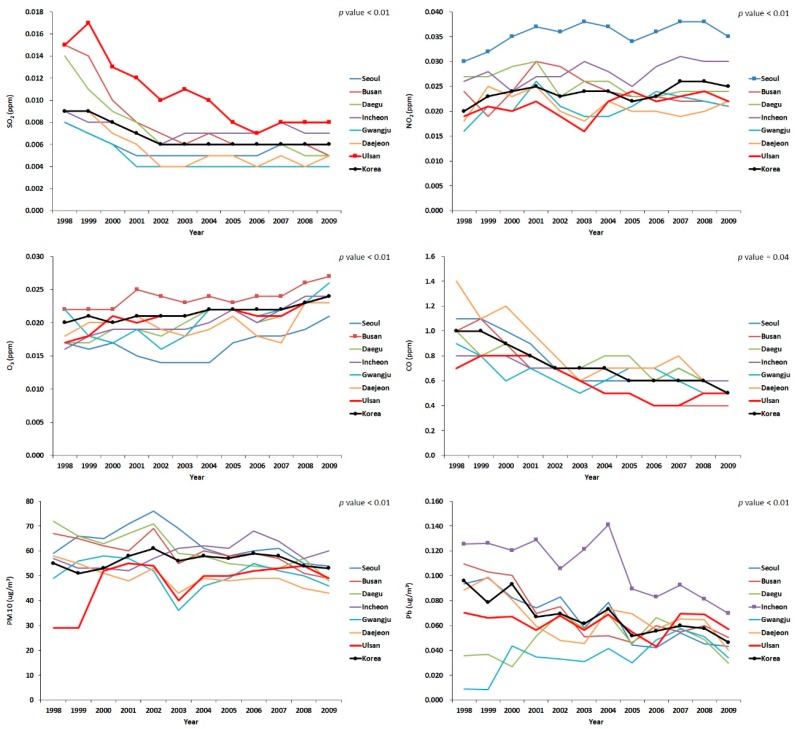
Annual averaged ambient concentrations of SO_2_, NO_2_, O_3_, CO, particulate matter 10 (PM10) and Pb between 1998 and 2009.

**Figure 2:**
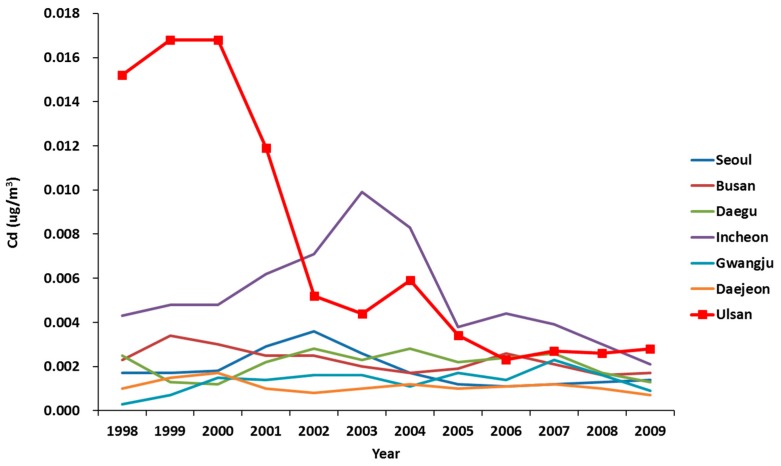
Annual averaged ambient concentrations of Cd between 1998 and 2009.

For atmospheric concentration, which is another indicator of air pollutant exposure, the six substances (SO_2_, NO_2_, O_3_, CO, PM10, Pb) from the air monitoring network data during the 1998–2009 period were identifiable for each target region. SO_2_ was the highest in Ulsan, NO_2_ was the highest in Seoul, O_3_ was the highest in Busan, and Pb was the highest in Incheon (Figure 1). From the atmospheric heavy metal monitoring networks, the concentration of Cd was prominently high in Ulsan before 2002 (Figure 2).

### 3.2. Smoking Rate

The smoking rates of all adults and males was the highest in Ulsan, and the values were 27.9% and 51.7%, respectively. For females, the smoking rate was the highest in Seoul at 4.0% and was the second highest in Incheon at 3.9%. The smoking rate of females in Ulsan and Daejeon were the third highest at 3.8% (Table 6).

### 3.3. Crude Rate, Age-Standardized Incidence Rate, Standardized Rate Ratio, and Adjusted Standardized Rate Ratio

During the 2000–2009 period, the ASR of lung cancer was the highest in all adults, males and females in Ulsan. The SRR was also the highest in all adults, males and females in Ulsan. The values were as follows: 1.091 (95% CI: 1.048–1.135), 1.131 (95% CI: 1.076–1.190) and 1.079 (95% CI: 1.001–1.163) respectively. The adjusted SRR was also the highest in all adults, males and females in Ulsan. These values were as follows: 1.152 (95% CI: 1.107–1.199), 1.487 (95% CI: 1.415–1.565) and 1.080 (95% CI: 1.002–1.164) respectively. The ASR of lung cancer was the second highest in all adults, males and females in Daegu, and both the SRR and adjusted SRR were significantly high. The SRR in all adults, males and females in Daegu were 1.051 (95% CI: 1.027–1.075), 1.053 (95% CI: 1.024–1.082) and 1.075 (95% CI: 1.029–1.122) respectively. The adjusted SRRs were as follows: 1.042 (95% CI: 1.018–1.066), 1.114 (95% CI: 1.084–1.145) and 1.064 (95% CI: 1.018–1.110) respectively. The adjusted SRRs in males in Busan and Incheon were significantly high, and the values were 1.208 (95% CI: 1.181–1.237) and 1.087 (95% CI: 1.056–1.118) respectively (Table 7).

## 4. Discussion

In Korea, the Ministry of Health and Welfare publishes a report annually, which shows cancer incidence by region, sex and age group. However, these results are yearly statistics and therefore the cancer incidence rate may fluctuate every year. It is difficult to obtain long-term stable cancer incidence data. The 5-year cumulative ASR provided by the KNSO complements the limitations of this annual report, but it still has limitations since it does not consider the causes of cancer. Therefore, in this study, the incidence of 10-year cumulative ASR of lung cancer during the 2000–2009 period was calculated to overcome these limitations and to compare the incidence of lung cancer among the seven metropolitan cities more precisely. Furthermore, occupational and environmental hazards were reviewed to examine the association between these factors and lung cancer.

Initially, we reviewed the NICs of the target regions. It was confirmed that the shipbuilding industry, automobile factories, non-ferrous metal, and iron and steel founding-related work, which are all associated with lung cancer as suggested by IARC, are concentrated in Ulsan. Petrochemical plants that process and produce various chemicals were also concentrated in Ulsan. In addition, air emissions of substances classified as IARC group 1, 2A and 2B were also found to be the highest in Ulsan. The average amount of emissions of such substances in Ulsan during the 2001–2009 period was approximately 4 times that of the second highest region, Daegu; 5 times that of the third highest region, Incheon; and 100 times that of Seoul, which has the lowest emissions. Therefore, based on the above results, workers in Ulsan may be exposed to more occupational substances related to lung cancer. For Incheon and Busan, the ratio of lung cancer-related industries (the shipbuilding industry, automobile factories, and the iron and steel founding industry) and petrochemical industries were higher than in other cities, and the results of the PRTR also showed that the emissions of carcinogenic substances in these cities are high. By substance, Daegu had the only asbestos air emissions until 2006, and before 2003 there was a particularly high emission rate. Busan was the only region with chromium atmospheric emissions during the 2001–2009 period, with the highest average emissions. Incheon showed the highest average emissions of nickel and the second highest average emissions of chromium.

Our analysis indicated that the adjusted SRR was significantly higher in males in Busan, Daegu, Incheon and Ulsan. This is important considering that the ratio of male to female workers in the non-ferrous metal industry, automobile factory, shipbuilding, and petrochemical industries is higher than in other industries [27]. In the cancer surveillance system of Korea, the incidence of lung cancer was high in manufacturing workers [28,29], and a study of male shipyard workers in Ulsan also showed that lung cancer incidence was significantly higher in blue-collar workers who were more likely to be exposed to lung cancer-related substances [30]. These studies support our findings.

Next, in order to examine the environmental hazards of the target area, we analyzed the data from the air pollution monitoring network. We found that the cadmium concentration was high in Ulsan before 2002. Studies on the relationship between environmental hazards and lung cancer have been conducted in the past, and many studies have reported a significant association between lung cancer and particulate matter 2.5 (PM2.5) due to air pollution [31]. Here, PM2.5 refers to small particles with an aerodynamic diameter of less than 2.5 μm, is composed of a mixture of various solid particles and aerosols, and is emitted mainly from vehicle exhaust, industrial facilities, and power plants. The criteria for such fine dusts are classified according to their size, and the composition may vary depending on the source and environmental factors [32]. Although the compositions of PM have not yet been clarified, past studies have shown that there is a difference in mutagenicity (capacity to cause mutations) depending on the day of the week [33], time of day [34], presence of urban/industrial facilities [35], altitude [36], and air pollutant concentration [37,38,39,40,41,42]. Especially in areas with urban and industrial facilities and areas with high concentrations of air pollutants, the mutagenicity of PM was higher. In Korea, the major components of PM2.5 were analyzed in 2009 in seven metropolitan cities. The highest concentrations of nickel, arsenic and cadmium (IARC group 1 substances), observed in PM2.5, were in Ulsan [18]. In Busan and Incheon, where the SRR and aSRR were significantly higher in males, the atmospheric concentration of nickel, arsenic and cadmium was higher than in other regions. Although studies on the composition of PM are limited, these results indicate that the composition of PM in urban and industrial areas may be different from that in other areas.

Based on these results, we think that the compositions of PM in the following regions are likely to be more mutagenic than in other regions: (1) Ulsan, which has the highest air emissions of carcinogenic substances, contains many industries associated with lung cancer, and has had a high concentration of atmospheric cadmium in the past; (2) Daegu, which has the second highest air emissions of carcinogenic substances and is the only region where asbestos air emissions exist; (3) Busan, which has a high level of carcinogenic emissions, has the highest level of chromium emissions and contains many industries associated with lung cancer; and (4) Incheon, which has a high level of emissions of carcinogenic substances, the highest air emissions of nickel, and contains many industries associated with lung cancer. In fact, the SRR and adjusted SRR were the highest in males and females in Ulsan, showing a statistically significant difference. The SRR and adjusted SRR were significantly elevated in Daegu in both males and females. The SRR and adjusted SRR were significantly elevated in males in Busan and Incheon, where the environmental hazards of lung cancer are relatively low.

In conclusion, based on the above results, occupational and environmental hazards may have an impact on lung cancer. This hypothesis is supported by the fact that the SRR and adjusted SRR of Ulsan, which has prominently higher occupational and environmental hazards compared to other regions, is significantly higher than that of the other regions in both males and females. In addition, the results of Daegu, Busan and Incheon support these findings.

Despite these significant results, there are some limitations to this study. First, we used CHS data to calculate the smoking rate since the Cancer Registry database did not present smoking history. Because the CHS was started in 2008, it was impossible to obtain the past smoking rates; therefore, we used the last year of the study period (2009) to represent the smoking rate. Since the smoking rate in Korea is now relatively low [2], the use of the 2009 smoking rate may underestimate the smoking rate of the past, which may result in overestimation of occupational and environmental factors. For the same reason, it is also a limitation that the smoking rate was indirectly adjusted, not directly. Second, this study cannot accurately identify occupational and environmental exposure prior to 1998, although the latency period of lung cancer is known to be at least 10 years [43]. Because we cannot obtain the data on occupational and environmental exposure, we assumed that environments were consistent over time, and conducted the analysis based on this assumption. Thus, the accuracy of the results may be somewhat reduced. Third, although occupational and environmental factors are known to be associated with the development of lung cancer, other factors such as genetic causes and past history of respiratory disease could not be considered in this study. Therefore, occupational and environmental factors may have been overestimated. Fourth, there may be a difference between the estimated amount of air pollutant exposure using data from the PRTR and Korean Ministry of Environment and the actual exposure of the subject. Fifth, if we had estimated the proportion of lung cancer cases that might be attributed to environmental/occupational exposure, the results of this study would show more information on the cause of lung cancer. However, we cannot estimate this due to the data limitations. It was difficult to calculate a single value for the relative risk for environmental and occupational exposures by combining the relative risks of lung cancer occurrence for each pollutant. Furthermore, the exposure data of all pollutants were not perfect by region. Finally, as this study is a cross-sectional study, we could evaluate the association between lung cancer and occupational and environmental factors, but could not evaluate causality.

Despite these limitations, this study is the first in Korea to analyze the 10-year cumulative ASR of lung cancer, and to examine the differences in ASR of lung cancer according to the occupational and environmental hazards of different regions. Moreover, although an indirect method was used, the smoking rate was adjusted. Therefore, we can conclude that the validity of the findings of this study is high, and the results of this study could be used as a good reference for future studies on lung cancer incidence.

## 5. Conclusions

Our findings revealed that lung cancer incidence was significantly elevated in the areas where occupational and environmental hazards were high. Over the past decades, many studies have been conducted on lung cancer, and many causes of lung cancer have been identified. However, to date, few studies have simultaneously considered the cumulative incidence, age standardization, smoking rate adjustment, and occupational and environmental hazards.

## Figures and Tables

**Table 1:** National industrial complex and major industries by region.

Region	National Industrial Complex (NIC)	Production Performance (Hundred Million Korean Won, %)						
		Petro-Chemistry	Nonferrous Metal	Iron and Steel	Machine	Electrical & Electronic	Transportation Equipment ^1^	Etc. ^2^
Seoul	Korea Export NIC	1039 (10.1)	87 (0.8)	79 (0.8)	1555 (15.2)	6067 (59.2)	80 (0.8)	1338 (13.1)
Busan	Myeongji Noksan NIC	515 (6.0)	98 (1.1)	1251 (14.6)	3619 (42.2)	1314 (15.3)	577 (6.7)	1195 (13.9)
Daegu	Daegu NIC	-	-	-	-	20 (9.8)	185 (90.2)	-
Incheon	Korea Export (Bupyeong) NIC	4493 (18.6)	110 (0.5)	1173 (4.8)	9707 (40.1)	4947 (20.4)	1889 (7.8)	1887 (7.8)
	Korea Export (Juan) NIC	192 (6.0)	1 (0.0)	42 (1.3)	1039 (32.3)	1417 (44.1)	34 (1.1)	489 (15.2)
	Namdong NIC	201 (5.2)	-	158 (4.1)	783 (20.2)	2014 (52.0)	539 (13.9)	178 (4.6)
Gwangju	Gwangju Science Valley	27 (0.6)	30 (0.6)	2 (0.0)	772 (16.4)	3606 (76.6)	273 (5.8)	-
Daejeon	Daedeok Science Town ^3^	-	-	-	-	-	-	-
Ulsan	Onsan NIC	16,272 (59.2)	100 (0.4)	8725 (31.8)	587 (2.1)	176 (0.6)	817 (3.0)	800 (2.9)
	Ulsan Mipo NIC	34,090 (37.2)	540 (0.6)	1337 (1.5)	2025 (2.2)	2240 (2.4)	50,598 (55.2)	817 (0.9)

Unit: Hundred Million Korean Won, %. ^1^ Transportation equipment: automobile, ship; ^2^ etc.: food, textile/clothing, wood/paper, etc.; ^3^ It is not an industrial complex but a research institute.

**Table 2:** Five-year cumulative Age-standardized incidence rate (ASR) of lung cancer by region.

Region	1999–2003 Year			2004–2008 Year			2009–2013 year		
	Total	Male	Female	Total	Male	Female	Total	Male	Female
Korea	28.5	50.8	12.9	29.2	50.1	14.3	28.7	46.6	15.4
Seoul	24.6	42.2	12.2	26.2	42.7	14.1	26.4	40.7	15.5
Busan	26.6	47.2	12.8	27.8	47.6	13.8	26.9	43.5	14.5
Daegu	29.8	52.4	14.3	30.4	52.3	15.2	30.2	49.6	16.2
Incheon	28.5	50.3	13.4	28	47.3	14.1	28.7	46.3	15.6
Gwangju	27.6	47.5	14.1	27.1	46.7	14	27.2	44.2	15
Daejeon	26.4	46.6	12.2	27.7	47.7	13.1	27.7	43.3	16.2
Ulsan	30.7	56.7	14.1	31.2	56.9	14	30.8	50.2	17.2

Unit: ASR per 100,000.

**Table 3:** Standard population of Korea.

Age Group (Years)	Korean Standard Population (Year 2000)
0–4	3,262,382
5–9	3,546,106
10–14	3,156,497
15–19	3,826,940
20–24	3,923,161
25–29	4,491,340
30–34	4,479,771
35–39	4,411,157
40–44	4,067,761
45–49	2,897,028
50–54	2,318,703
55–59	2,088,513
60–64	1,796,705
65–69	1,301,094
70–74	883,475
75–79	587,065
80–84	309,500
85+	186,926
Total	47,534,124

Unit: persons.

**Table 4:** Annual emissions of air pollutants* among metropolitan cities in Korea during the 2001–2009 period.

Region	2001	2002	2003	2004	2005	2006	2007	2008	2009	Mean
Seoul	524	1469	2270	39,068	36,394	29,791	19,601	1650	1302	14,674
Busan	10,885	82,805	56,553	179,386	145,786	174,994	244,136	162,072	84,657	126,808
Daegu	174,517	464,850	248,844	496,604	235,056	253,546	260,998	216,080	218,502	285,444
Incheon	237,535	261,416	154,653	331,687	325,806	282,128	289,086	246,618	119,434	249,818
Gwangju	162,736	160,038	17,413	118,279	140,370	694,888	514,432	132,064	148,642	232,096
Daejeon	4161	4487	8554	108,001	103,691	131,326	99,042	109,223	59,697	69,798
Ulsan	1,693,133	1,325,417	1,086,807	1,097,231	1,303,247	921,108	1,011,435	972,537	1,035,342	1,160,695

Unit: kg/year. * Includes International Agency for Research on Cancer (IARC) group 1 (human carcinogen), group 2A (probably human carcinogen), and group 2B (possibly human carcinogen) substances.

**Table 5:** Annual emissions of asbestos, arsenic, chromium and nickel among metropolitan cities in Korea during the 2001–2009 period.

	Region	2001	2002	2003	2004	2005	2006	2007	2008	2009	Mean
Asbestos	Seoul	-	-	-	-	-	-	-	-	-	-
	Busan	-	-	-	-	-	-	-	-	-	-
	Daegu	3627	5919	537	96	62	39	-	-	-	1142
	Incheon	-	-	-	-	-	-	-	-	-	-
	Gwangju	-	-	-	-	-	-	-	-	-	-
	Daejeon	-	-	-	-	-	-	-	-	-	-
	Ulsan	-	-	-	-	-	-	-	-	-	-
Arsenic	Seoul	-	-	-	-	-	-	-	-	-	-
	Busan	-	-	-	-	-	-	-	-	-	-
	Daegu	-	-	-	-	-	-	-	-	-	-
	Incheon	-	-	-	-	-	-	-	-	-	-
	Gwangju	-	-	-	-	-	-	-	-	-	-
	Daejeon	-	-	-	-	-	-	-	-	-	-
	Ulsan	-	-	-	-	24	31	29	36	22	16
Chromium	Seoul	-	-	-	-	-	-	-	-	-	-
	Busan	475	748	456	1697	2,329	341	127	153	434	751
	Daegu	-	-	2	63	133	302	105	84	78	85
	Incheon	-	-	9	612	668	2403	1391	454	637	686
	Gwangju	-	-	-	-	2	7	33	29	9	9
	Daejeon	-	-	-	16	16	227	841	765	278	238
	Ulsan	-	-	1	45	103	56	6	95	10	35
Nickel	Seoul	-	-	-	212	245	338	63	437	222	169
	Busan	-	2	-	184	221	84	61	70	75	77
	Daegu	-	-	-	524	237	177	189	239	31	155
	Incheon	-	1362	21	7173	7207	272	328	246	177	1865
	Gwangju	-	-	-	136	128	139	433	455	316	179
	Daejeon	-	-	-	-	-	-	-	-	-	-
	Ulsan	-	-	-	16	30	38	18	11	1	13

Unit: kg/year.

**Table 6:** Smoking rate (%) by region in 2009.

Region	Total	Male	Female
Korea	26.0	48.7	3.7
Seoul	24.3	45.8	4.0
Busan	27.1	51.5	3.7
Daegu	25.7	49.4	3.1
Incheon	26.8	50.0	3.9
Gwangju	22.1	43.3	2.1
Daejeon	25.5	48.1	3.8
Ulsan	27.9	51.7	3.8

**Table 7:** SRR and aSRR of lung cancer incidence among metropolitan cities in Korea during the 2000–2009 period.

	Region	Number	CR ^1^	ASR ^2^	SRR ^3^	95% CI ^4^	aSRR ^5^	95% CI
Total	Korea	165,894	34.1	28.7	1.000		1.000	
	Seoul	27,442	26.9	25.5	0.886	0.876–0.897	0.846	0.836–0.856
	Busan	11,703	31.9	27	0.940	0.923–0.957	0.969	0.951–0.986
	Daegu	8213	32.7	30.2	1.051	1.027–1.075	1.042	1.018–1.066
	Incheon	7167	27.5	28.2	0.981	0.958–1.004	1.002	0.979–1.026
	Gwangju	3807	27.2	27.3	0.951	0.921–0.981	0.856	0.829–0.883
	Daejeon	3834	26.7	27.3	0.949	0.920–0.980	0.936	0.907–0.967
	Ulsan	2746	25.5	31.3	1.091	1.048–1.135	1.152	1.107–1.199
Male	Korea	120,265	49.3	50.1	1.000		1.000	
	Seoul	19,291	37.9	42.3	0.845	0.833–0.858	0.684	0.675–0.695
	Busan	8425	46.0	46.9	0.937	0.916–0.959	1.208	1.181–1.237
	Daegu	5858	46.5	52.7	1.053	1.024–1.082	1.114	1.084–1.145
	Incheon	5154	39.3	48.7	0.972	0.945–1.000	1.087	1.056–1.118
	Gwangju	2667	38.4	47.2	0.943	0.907-0.979	0.657	0.632–0.682
	Daejeon	2770	38.4	47.3	0.945	0.910–0.981	0.898	0.865–0.933
	Ulsan	1982	35.8	56.6	1.131	1.076–1.190	1.487	1.415–1.565
Female	Korea	45,629	18.8	13.7	1.000		1.000	
	Seoul	8151	15.9	13.3	0.974	0.951–0.997	0.978	0.955–1.002
	Busan	3278	17.8	13.2	0.962	0.929–0.996	0.961	0.928–0.995
	Daegu	2355	18.8	14.7	1.075	1.029–1.122	1.064	1.018–1.110
	Incheon	2013	15.6	13.6	0.995	0.951–1.041	0.998	0.953–1.044
	Gwangju	1140	16.2	13.9	1.017	0.957–1.080	0.990	0.931–1.051
	Daejeon	1064	14.8	13.0	0.953	0.897–1.012	0.954	0.898–1.013
	Ulsan	764	14.6	14.8	1.079	1.001–1.163	1.080	1.002–1.164

Units: persons; CR per 100,000; ASR per 100,000. ^1^ CR, crude ratio; ^2^ ASR, age-standardized incidence rate; ^3^ SRR, standardized rate ratio; ^4^ 95% CI, 95% confidence interval; ^5^ aSRR, adjusted standardized rate ratio.
